# Molecular Regulation of Transforming Growth Factor-β1-induced Thioredoxin-interacting Protein Ubiquitination and Proteasomal Degradation in Lung Fibroblasts: Implication in Pulmonary Fibrosis

**DOI:** 10.35534/jrbtm.2024.10002

**Published:** 2024-02-01

**Authors:** Sarah J Taleb, Qinmao Ye, Boina Baoyinna, Michael Dedad, Dakshin Pisini, Narasimham L Parinandi, Lewis C Cantley, Jing Zhao, Yutong Zhao

**Affiliations:** 1 Department of Physiology and Cell Biology, Dorothy M. Davis Heart and Lung Research Institute, The Ohio State University, Columbus, OH, USA; 2 Department of Internal Medicine, The Ohio State University, Columbus, OH, USA; 3 Dana-Farber Cancer Institute, Harvard Medical School, Boston, MA, USA

**Keywords:** TXNIP, Lung fibroblasts, Lung fibrosis, Deubiquitination, TGF-β1, USP13

## Abstract

Thioredoxin-interacting protein (TXNIP) plays a critical role in regulation of cellular redox reactions and inflammatory responses by interacting with thioredoxin (TRX) or the inflammasome. The role of TXNIP in lung fibrosis and molecular regulation of its stability have not been well studied. Therefore, here we investigated the molecular regulation of TXNIP stability and its role in TGF-β1-mediated signaling in lung fibroblasts. TXNIP protein levels were significantly decreased in lung tissues from bleomycin-challenged mice. Overexpression of TXNIP attenuated transforming growth factor-β1 (TGF-β1)-induced phosphorylation of Smad2/3 and fibronectin expression in lung fibroblasts, suggesting that decrease in TXNIP may contribute to the pathogenesis of lung fibrosis. Further, we observed that TGF-β1 lowered TXNIP protein levels, while *TXNIP* mRNA levels were unaltered by TGF-β1 exposure. TGF-β1 induced TXNIP degradation via the ubiquitin-proteasome system. A serine residue mutant (TNXIP-S308A) was resistant to TGF-β1-induced degradation. Furthermore, downregulationof ubiquitin-specific protease-13 (USP13) promoted the TGF-β1-induced TXNIP ubiquitination and degradation. Mechanistic studies revealed that USP13 targeted and deubiquitinated TXNIP. The results of this study revealed that the decrease of TXNIP in lungs apparently contributes to the pathogenesis of pulmonary fibrosis and that USP13 can target TXNP for deubiquitination and regulate its stability.

## 1. Introduction

Pulmonary fibrosis is a chronic lung disease characterized by extracellular matrix accumulation and impaired gas exchange. Uncontrolled repair and remodeling after repeated lung injury induces excessive fibroblast to myofibroblast differentiation. Currently, there is no effective therapy to cure pulmonary fibrosis [1-3]. Transforming growth factor-β1 (TGF-β1) is a major cytokine involved in activation of fibroblasts and their differentiation to myofibroblasts during the development of fibrosis. TGF-β1 binds to TGF-β1 receptors and activates the canonical signaling pathway via SMAD2/3, as well as non-canonical signaling, such as phosphorylation of p38 MAPK [4,5]. Understanding the TGF-β1-mediated signaling pathway is vital for development of therapies to prevent and ameliorate pulmonary fibrosis.

Ubiquitination is a protein post-translational modification that regulates protein stability, protein-protein interaction, intracellular translocation, and enzyme activity. Ubiquitin E3 ligases and deubiquitinases (DUBs) mediate and balance protein ubiquitination levels [6,7]. One of our recent studies showed that TGF-β1 regulates protein degradation by reducing E3 ligase *Nedd4L* gene expression in lung fibroblasts [8]. However, the direct effect of TGF-β1-mediated signaling on protein degradation has not been well studied.

Thioredoxin-interacting protein (TXNIP) has been identified as a thioredoxin (TRX) binding protein and inhibits its antioxidant function [9,10]. It has been shown to play a critical role in regulation of oxidative stress [11,12]. Recent studies suggest that TXNIP participates in inflammatory responses by interacting with the NLRP3 inflammasome [13]. TXNIP has also been shown to regulate gene expression through the TXNIP-HIF1α-TWIST signaling axis or by direct interaction with transcriptional factors [14]. Ubiquitination-mediated regulation of TXNIP stability has been reported. Itch E3 ligase has been found to ubiquitinate TXNIP and induce its proteasomal degradation [15,16]. However, the DUB for TXNIP has not been well investigated.

To understand the role of TXNIP in pulmonary fibrosis, we focused on investigating TXNIP’s stability in lung fibroblasts. We showed that TXNIP levels were reduced in bleomycin-induced lung fibrosis and in TGF-β1-treated lung fibroblasts and epithelial cells. Overexpression of TXNIP reduced TGF-β1-mediated phosphorylation of Smad2/3 and fibronectin expression. Further, we found that USP13, a DUB, stabilized TXNIP in lung fibroblasts. This study underpins the anti-fibrotic property of TXNIP in pulmonary fibrosis through attenuation of TGF-β1 signaling and emphasizes that TXNIP is unstable under TGF-β1 treatment, but can be stabilized by USP13.

## 2. Materials and Methods

### 2.1. Animal Experiments

C57BL/6J mice (8–10 weeks, Strain #000064) were purchased from Jackson Laboratory and housed in the specific pathogen-free animal care facility at the Ohio State University in accordance with institutional guidelines and guidelines of the US National Institutes of Health. All animal experiments were approved by an institutional animal care and use committee (IACUC) at the Ohio State University Animal Resources Centers. Mice were challenged with a single intratracheal instillation of bleomycin (BLM, 0.045 units per mouse) or PBS. After 21 days, lungs were harvested for further analysis.

### 2.2. Cell Culture and Reagents

Human lung fibroblast cell line (IMR90) cells were purchased from ATCC (Manassas, VA, USA) and cultured in EMEM (Gibco, Billings, Montana) supplemented with 10% fetal bovine serum (FBS) and 1% penicillin/streptomycin. A549 and MLE12 (mouse lung epithelial) cells were purchased from ATCC and cultured in RPMI-1649 (Gibco) supplemented with 10% fetal bovine serum (FBS) and 1% penicillin/streptomycin. Cells were maintained in a 37 °C incubator under a humidified atmosphere of of 5% CO_2_. Recombinant human TGF-β1 and BMP4 were purchased from R&D systems (Minneapolis, MN, USA). CRM1 inhibitor III (CRM1i) and MG-132 were purchased MilliporeSigma (Burlington, MA, USA). Anti-HO-1 antibody was purchased from Abcam (Cambridge, UK). Immobilized protein A/G beads, anti-V5, and anti-GAPDH antibodies were purchased from Santa Cruz Biotechnology (Santa Cruz, CA, USA). Anti-USP13 and anti-TXNIP antibodies were purchased from ProteinTech (Chicago, IL, USA). Anti-HA tag and anti-β-actin antibodies, cycloheximide (CHX), and leupeptin were purchased from Sigma-Aldrich (St. Louis, MO, USA). Anti-collagen1a1, anti-fibronectin (FN), anti-Smad3, and anti-p-Smad2/3 antibodies were purchased from Cell Signaling Technology (Danvers, MA, USA). Horseradish peroxidase-conjugated goat anti-rabbit and anti-mouse secondary antibodies were purchased from Bio-Rad Laboratories (Hercules, CA, USA). All commercially available materials used were of the highest available quality.

### 2.3. Plasmid and siRNA Transfection

Human *USP13* siRNA and control siRNA were purchased from Sigma Aldrich (St Louis, MO, USA). Cells were cultured on 60-mm dishes to 70–90% confluence. Plasmids encoding HA-tagged human TXNIP and TXNIP-S308A, empty vector, and siRNAs were transfected into IMR90 cells using PepMute^™^ siRNA transfection reagent or GenJet^™^ DNA transfection reagents (SignaGen, Rockville, MD) according to the transfection reagent manufacturer’s instructions.

### 2.4. Western Blotting Analysis

An equal amount of cell lysates (10–20 μg) was loaded for the SDS-PAGE protein separation. The proteins were separated on the gel and then transferred to membranes. Immunoblotting was performed with incubation with primary and secondary antibodies according to the laboratory’s standard protocol [17].

### 2.5. qRT-PCR Analysis

Total RNA was isolated from cultured IMR90 cells using the Total RNA Mini kit (IBI Scientific, IA, USA) according to the manufacturer’s instructions. cDNA was prepared using the iScript cDNA synthesis kit (Bio-Rad, CA, USA). The expression of *FN* and *TXNIP* were performed using iQ SYBR Green Supermix and the iCycler real-time PCR detection system (Bio-Rad, CA, USA). *GAPDH* was used as an internal control. *FN* primers: forward, GGTCCGGGACTCAATCCAAA, reverse, GACAGAGTTGCCCACGGTAA; TXNIP primers: forward, CTTAGTGTAACCAGCGGCGT, reverse, CTGAGGAAGCTCAAAGCCGA; *GAPDH* primers: forward, TCGGAGTCAACGGATTTGGTCG, reverse, GCTCTCCAGAACATCATCCCTGCCT.

### 2.6. Co-immunofluorescence Staining

IMR90 cells were cultured in glass-bottom dishes. Cells were fixed with 3.7% formaldehyde for 20 min. After blocking in 1% bovine serum albumin in Tris-buffered saline with 0.1% Tween-20 for 1 h, cells were incubated with 1:200 dilution of primary antibodies for 1 h, followed by a 1:200 dilution of fluorescence-conjugated secondary antibodies for immunostaining. Images were captured by Nikon A1R-HD25 confocal microscope (Nikon, Tokyo, Japan).

### 2.7. In Vitro Ubiquitination Assay

A modified protocol under denaturing conditions was used to determine TXNIP ubiquitination. HEK293 cells were transfected with HA-tagged ubituitin plasmids, then after 48 h treated with MG-132 for 1 h followed by TGF-β1 for 3 h. Cells were harvested in PBS and then subjected to centrifugation at 4000 rpm for 5 min, after which the supernatant was discarded. The cell pellets were resuspended in 1 μL of ubiquitin aldehyde, 1 μL of NEM and 70 μL of 2% SDS lysis buffer. The samples were diluted with 700 μL of 1 × TBS after sonication (12 s). Equal amounts of protein (1 mg) were incubated with the anti-TXNIP, ubiquitin antibody, or IgG overnight at 4 °C. 40 μL protein A/G plus agarose beads were added and samples were incubated for an additional 2 h at 4 °C. The immunoprecipitated complex was washed three times with cold PBS and analyzed by immunoblotting with antibodies against HA tag or TXNIP.

### 2.8. Quantification and Statistical Analysis

Immunoblot intensities were quantified by ImageJ software (Image Processing and Analysis in Java; National Institutes of Health, Bethesda, MD, USA; http://imagej.nih.gov/). One-way ANOVA statistical analysis was used for comparison among several groups. Data are expressed as mean ± SEM of triplicate samples from at least three independent experiments. *p* < 0.05 were considered statistically significant.

## 3. Results

### 3.1. TXNIP Protein Levels Were Reduced in Fibrotic Lungs

**Figure 1.**
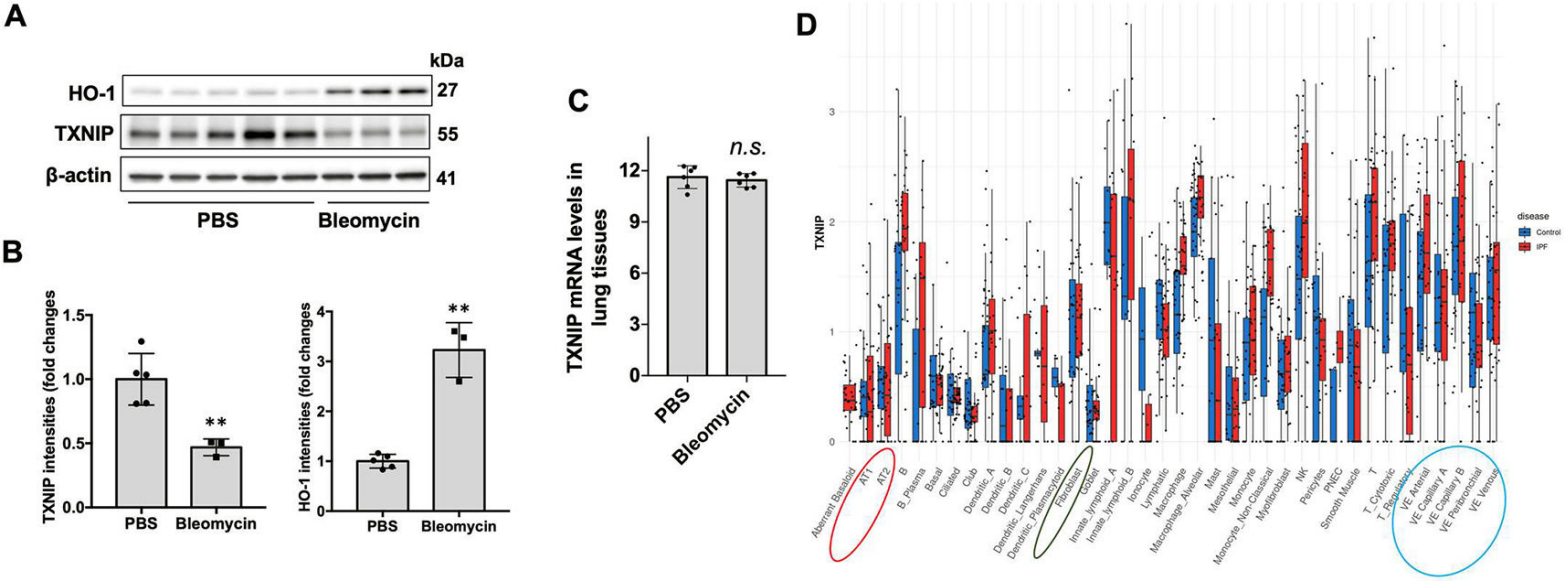
TXNIP levels were lower in lung tissues of bleomycin-challenged mice. (**A**) Male C57/BL6J mice were intratracheally challenged with PBS or bleomycin (0.045 U/mouse). After 3 weeks, lung tissue lysates were analyzed by immunoblotting with HO-1, TXNIP, and β-actin antibodies. (**B**) Intensities of TXNIP and HO-1 were quantified by ImageJ software. *n* = 3–5. **p < 0.01 compared to PBS-treated mice. (**C**) Re-anaysis of TXNIP gene transcript in GDS4902 and GDS5078 databases. (**D**) Single cell RNAseq analysis of gene expression of TXNIP in lung cells from control (cont) and IPF patients (Kaminski/Rosas data base from ipfcellatlas.com). Red circle: alveolar epithelial cells; black circle: fibroblasts; blue circle: endothelial cells.

TXNIP plays multiple roles in various cellular responses by regulating reactive oxygen species (ROS) generation, NLRP3 inflammasome activation, and gene expression [11-14]. We have shown that heme oxygenase-1 (HO-1), an antioxidant, was increased in fibrotic lungs in a bleomycin-induced murine model of lung fibrosis [18]. TXNIP has been considered as a pro-oxidant and displays an opposite effect than that of HO-1 [19]. To investigate the role of TXNIP in the pathogenesis of lung fibrosis, we examined the TXNIP levels in lung tissues from PBS and bleomycin-challenged mice. Bleomycin induced HO-1 levels, which is consistent with our previous report [18]. Interestingly, TXNIP levels were significantly lower in fibrotic lungs (Figure 1A,B). Re-analysis of GDS4902 and GDS5078 microarray databases indicates no signfinicant changes in TXNIP mRNA levels in bleomcyin-induced lung fibrosis model (Figure 1C). Furthermore, gene expression profiles of lungs from normal subjects compared to those from idiopathic pulmonary fibrosis (IPF) patients (Kaminski/Rosas data base from ipfcellatlas.com) did not indicate a difference in TXNIP gene expression levels in alveolar epithelial cells (AT1 and AT2), vascular endothelial cells, or fibroblasts (Figure 1D), suggesting that the changes of TXNIP levels in fibrotic lungs may occur due to protein degradation.

### 3.2. TXNIP Attenuates TGF-β1-induced Profibrotic Effects in Lung Fibroblasts

**Figure 2.**
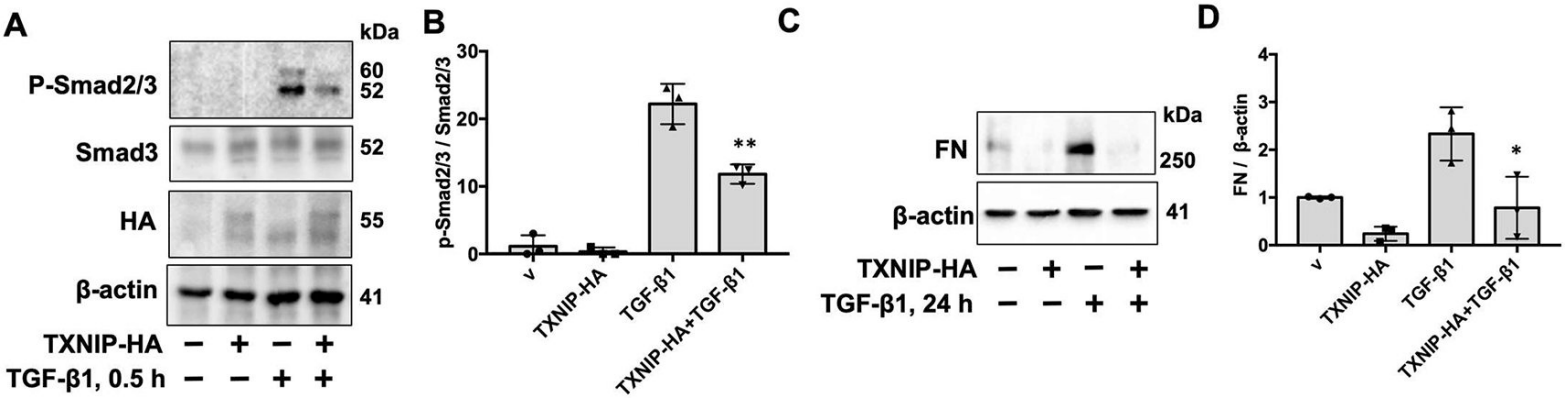
Overexpression of TXNIP attenuates TGF-β1-induced phosphorylation of Smad2/3 and FN expression in lung fibroblasts. (**A**) IMR90 cells were transfected with plasmid encoding HA-tagged TXNIP (TXNIP-HA) for 48 h, then treated with TGF-β1 (10 ng/mL) for 0.5 h. Cell lysates were analyzed by immunoblotting with phopho (p)-Smad2/3, Smad3, HA tag, and β-actin antibodies. (**B**) Intensities of p-Smad2/3 were quantified by ImageJ software and normalized to Smad2/3. *n* = 3. **p < 0.01 compared to TGF-β1 alone. (**C**) IMR90 cells were transfected with plasmid encoding HA-tagged TXNIP (TXNIP-HA) for 48 h, then treated with TGF-β1 (10 ng/mL) for 24 h. Cell lysates were analyzed by immunoblotting with FN and β-actin antibodies. (**D**) Intensities of FN were quantified by ImageJ software and normalized to β-actin. *n* = 3. *p < 0.05 compared to TGF-β1 alone. Representative blots from three independent experiments are shown.

To investigate if the reduction of TXNIP contributes to the pathogenesis of lung fibrosis, we first measured ROS levels in TXNIP-overexpressed lung fibroblasts. Though it has been shown that TXNIP functions as a pro-oxidant, we did not detect ROS changes in TXNIP-overexpressed lung fibroblasts (Figure S1). TGF-β1 is a major cytokine that induces fibroblast to myofibroblast differentiation and extracellular matrix (ECM) accumulation. Therefore, we examined the effect of TXNIP on the TGF-β1-induced signaling pathway. Overexpression of TXNIP attenuated TGF-β1-induced phosphorylation of Smad2/3, without altering total Smad2/3 levels (Figure 2A,B). This phenomenon was also observed in lung epithelial cells (Figure S2A,B). TGF-β1 treatment for 24 h increased FN levels, but this effect decreased in TXNIP-overexpressed lung fibroblasts (Figure 2C,D). These results suggest that TXNIP exhibits an anti-fibrotic property by suppressing the actions of TGF-β1. Thus, the reduction of TXNIP in lungs may promote fibrotic responses and contribute to the development of lung fibrosis.

### 3.3. TGF-β1 Treatment Induces TXNIP Degradation

**Figure 3.**
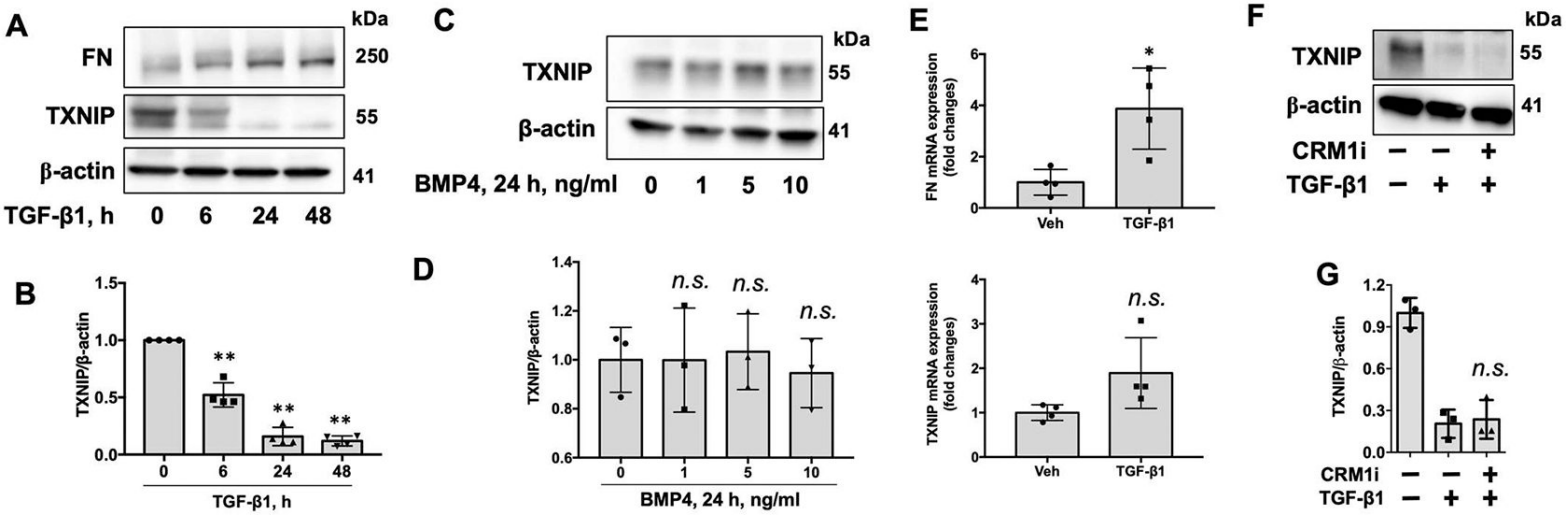
TGF-β1 treatment lowers TXNIP protein levels, not mRNA levels. (**A**) IMR90 cells were treated with TGF-β1 (10 ng/mL) for 0–48 h. Cell lysates were analyzed by immunoblotting with FN, TXNIP, and β-actin antibodies. (**B**) Intensities of TXNIP were quantified by ImageJ software and normalized to β-actin. *n* = 3. ** *p* < 0.01 compared to 0 h. (**C**) IMR90 cells were treated with BMP4 (0–10 ng/mL) for 24 h. Cell lysates were analyzed by immunoblotting with TXNIP and β-actin antibodies. (**D**) Intensities of TXNIP were quantified by ImageJ software and normalized to β-actin. *n* = 3. n.s. *p* > 0.05 compared to 0 ng/mL. (**E**) IMR90 cells were treated with TGF-β1 (10 ng/mL) for 24 h, and total RNA was extracted. *FN* and *TXNIP* mRNA levels were measured by realtime PCR and normalized to *GAPDH* mRNA levels. *n* = 4. *p < 0.05, n.s. *p* > 0.05 compared to vehicle (Veh). (**F**) IMR90 cells were treated with CRM1 inhibitor III (CRM1i, 10 μM) for 1 h prior to TGF-β1 (10 ng/mL) treatment for 6 h. Cell lysates were analyzed by immunoblotting with TXNIP and β-actin antibodies. **G.** Intensities of TXNIP were quantified by ImageJ software and normalized to β-actin. *n* = 3. n.s. *p* > 0.05 compared to TGF-β1 alone. Representative blots from three independent experiments are shown.

As we demonstrated that reduction of TXNIP may contribute to pathogenesis of lung fibrosis, we further investigated the molecular regulation of TXNIP degradation. As TGF-β1 is important in development of lung fibrosis, we examined TXNIP levels under TGF-β1 treatment. As shown in Figure 3A,B, TGF-β1 treatment decreased TXNIP levels in lung fibroblasts. The increased FN levels confirmed the bioactivity of TGF-β1 in the same experiment. To investigate if this reduction in TXNIP is specifically triggered by TGF-β1, we examined the TXNIP levels after treatment with an anti-fibrotic cytokine, bone morphogenetic protein 4 (BMP4), which belongs to the TGF superfamily. BMP4 treatment for 24 h had no effects on TXNIP levels (Figure 3C,D), suggesting that TGF-β1, a profibrotic cytokine, induces TXNIP decrease. Further, we found that *TXNIP* mRNA levels were not altered by TGF-β1. *FN* mRNA levels were increased by TGF-β1, as a positive control for TGF-β1 treatment (Figure 3E). These data suggest that the decrease of TXNIP protein levels in the presence of TGF-β1 is caused by protein degradation. TXNIP is exclusively localized in the nucleus. However, inhibiting CRM1-mediated nuclear export did not attenuate TGF-β1-induced TXNIP degradation (Figure 3F,G), suggesting that TGF-β1 induces TXNIP degradation in the nucleus.

### 3.4. TGF-β1 Induces TXNIP Ubiquitination and Proteasomal Degradation

**Figure 4.**
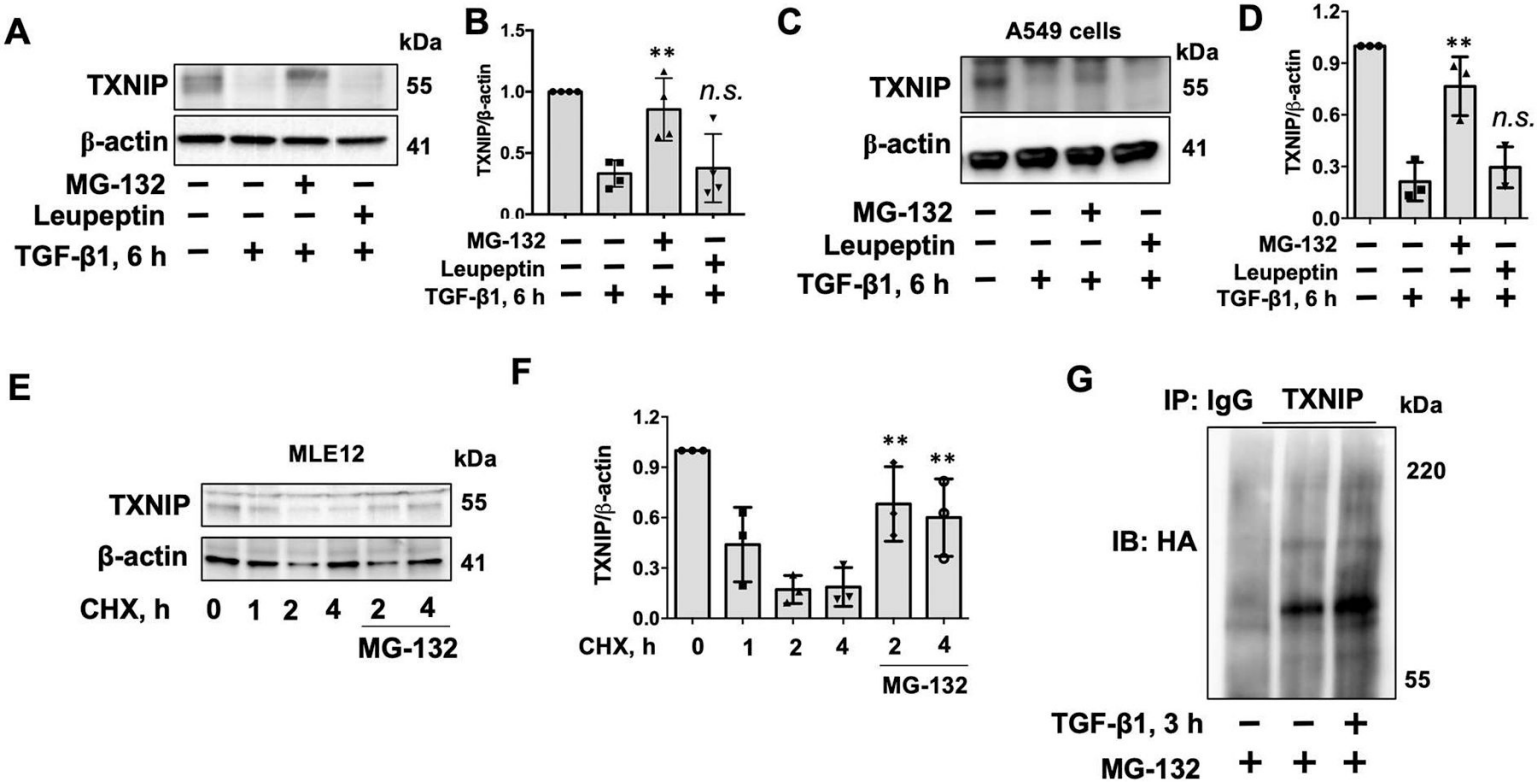
TGF-β1 induces TXNIP degradation in the ubiquitin-proteasome system. (**A**) IMR90 cells were treated with MG-132 (20 μM) or leupeptin (100 μM) for 1 h, and then challenged with TGF-β1 (10 ng/mL) for additional 6 h. Cell lysates were analyzed by immunoblotting with TXNIP and β-actin antibodies. (**B**) Intensities of TXNIP were quantified by ImageJ software and normalized to β-actin. *n* = 4. ** *p* < 0.01, n.s. *p* > 0.05 compared to TGF-β1 alone. (**C**) A549 cells were treated with MG-132 (20 μM) or leupeptin (100 μM) for 1 h, and then challenged with TGF-β1 (10 ng/mL) for additional 6 h. Cell lysates were analyzed by immunoblotting with TXNIP and β-actin antibodies. (**D**) Intensities of TXNIP were quantified by ImageJ software and normalized to β-actin. *n* = 3. ** *p* < 0.01, n.s. *p* > 0.05 compared to TGF-β1 alone. (**E**) MLE12 cells were treated with MG-132 (20 μM) for 1 h, then challenged with CHX (20 ng/mL) for additional 0–4 h. Cell lysates were analyzed by immunoblotting with TXNIP and β-actin antibodies. (**F**) Intensities of TXNIP were quantified by ImageJ software and normalized to β-actin. *n* = 3. ** *p* < 0.01 compared to CHX 2 and 4 h. Shown are representative blots from three to four independent experiments. (**G**) HEK293 cells were transfected with plasmids encoding HA-tagged ubiquitin for 48 h, and then treated with MG-132 (20 μM) for 1 h prior to TGF-β1 treatment (10 ng/mL, 3 h). Denatured cell lysates were subjected to immunoprecipitation with rabbit IgG and TXNIP antibody, followed by immunoblotting with HA tag antibody.

**Figure 5.**
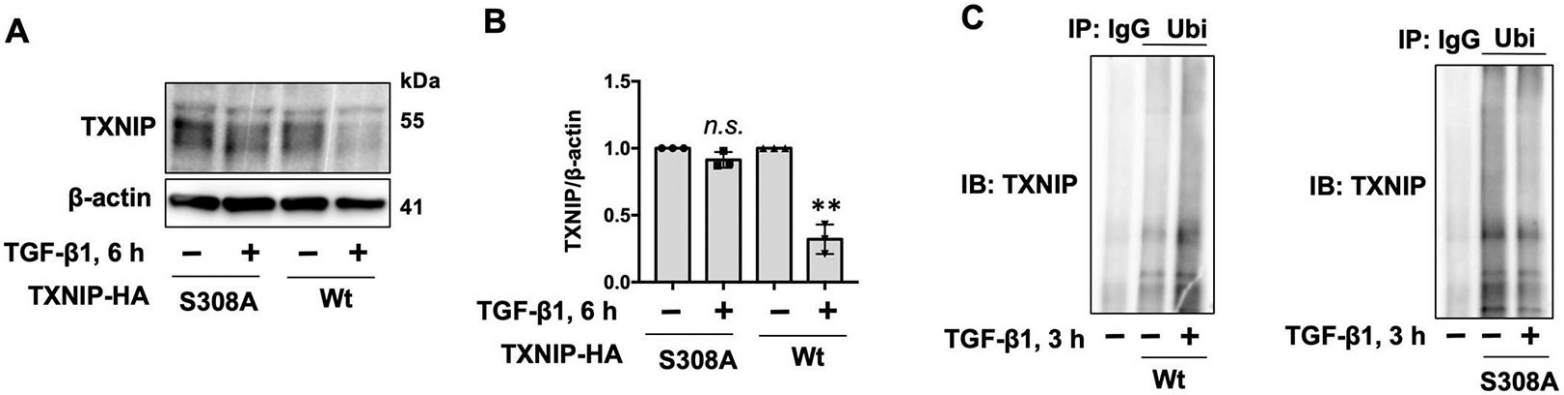
Ser 308 is critical for TGF-β1-induced TXNIP degradation. (**A**) HEK293 cells were transfected with plasmids encoding TXNIP-HA or TXNIP-S308A-HA for 48 h, and then cells were treated with TGF-β1 (10 ng/mL) for additional 6 h. Cell lysates were analyzed by immunoblotting with TXNIP and β-actin antibodies. (**B**) Intensities of TXNIP were quantified by ImageJ software and normalized to β-actin. *n* = 3. ** *p* < 0.01, compared to Wt alone; n.s. *p* > 0.05 compared to S308A alone. Shown are representative blots from three independent experiments. (**C**) HEK293 cells were transfected with plasmids encoding TXNIP-HA or TXNIP-S308A-HA for 48 h, and then treated with MG-132 (20 μM) for 1 h prior to TGF-β1 treatment (10 ng/mL, 3 h). Denatured cell lysates were subjected to immunoprecipitation with rabbit IgG and ubiquitin (Ubi) antibody, followed by immunoblotting with TXNIP antibody.

Protein degradation occurs mainly in the proteasome and lysosome. TGF-β1-induced TXNIP degradation was attenuated by a proteasome inhibitor (MG-132), but not a lysosome inhibitor (leupeptin) in lung fibroblasts (Figure 4A,B), human lung epithelial cells (Figure 4C,D), and mouse lung epithelial cells (Figure 4E,F), indicating that TGF-β1-induced TXNIP degradation occurs in the proteasome. Ubiquitination is a post-translational modification that often promotes proteasomal degradation of proteins. TGF-β1 treatment increased TXNIP polyubiquitination (Figure 4G), suggesting that TGF-β1 induces TXNIP ubiquitination and proteasomal degradation. Phosphorylation of TXNIP at serine (Ser) residue 308 has been reported to promote TXNIP degradation [20]. To investigate if the serine (ser) 308 residue is critical for TGF-β1-induced TXNIP degradation, we overexpressed HA tagged TXNIP wild type and TXNIP mutant (S308A) in HEK293 cells, which were then treated with TGF-β1 for 6 h. As shown in Figure 5A,B, the S308A mutant demonstrated resistance to TGF-β1-induced degradation compared to wild type TXNIP. Further, TGF-β1 increased polyubiqutination of TXNIP wild type, but not the S308A mutant (Figure 5C), suggesting that the ser 308 residue plays a critical role in TGF-β1-induced TXNIP ubiquitination and degradation.

### 3.5. USP13 Deubiqitinates and Stabilizes TXNIP

**Figure 6.**
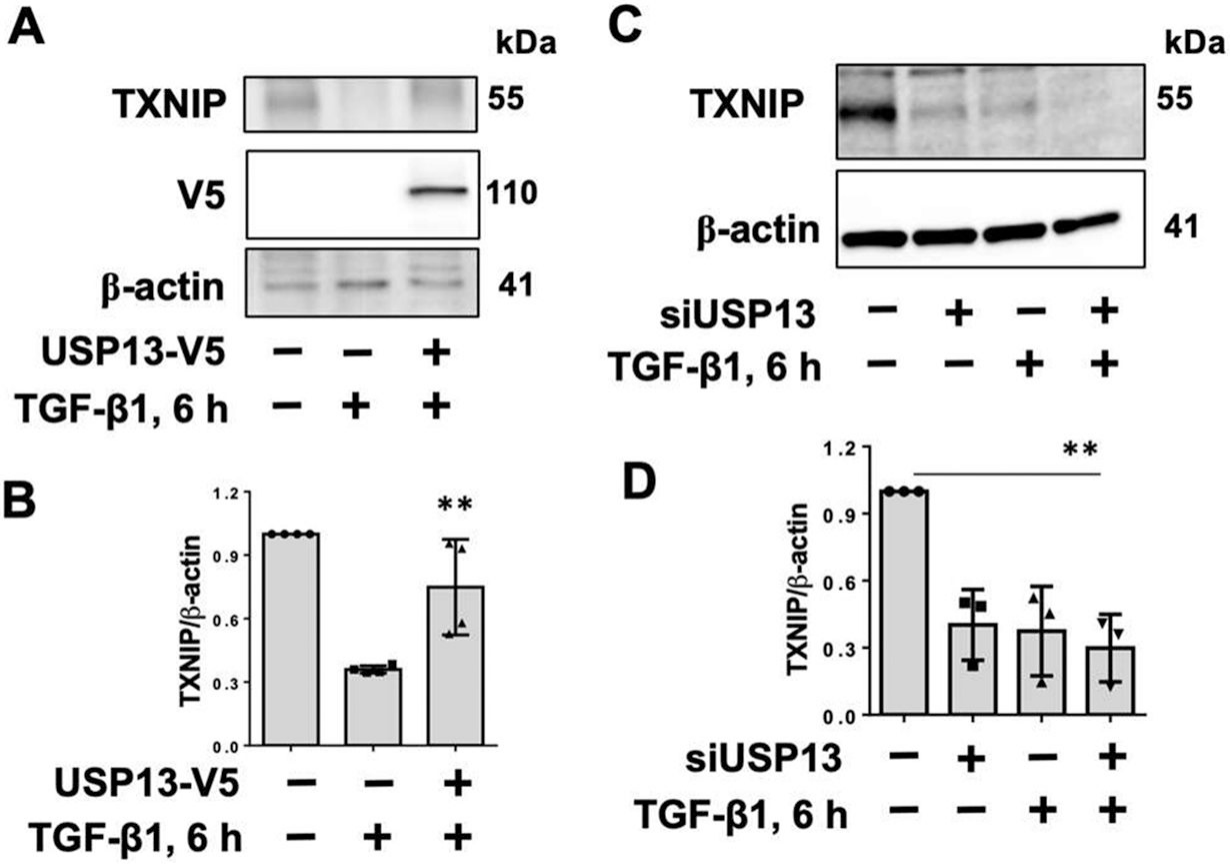
USP13 stabilizes TXNIP. (**A**) IMR90 cells were transfected with plasmids encoding V5-tagged USP13 for 48 h, and then treated with TGF-β1 (10 ng/mL) for additional 6 h. Cell lysates were analyzed by immunoblotting with TXNIP, V5 tag, and β-actin antibodies. (**B**) Intensities of TXNIP were quantified by ImageJ software and normalized to β-actin. *n* = 3. ** *p* < 0.01, compared to TGF-β1 alone. (**C**) IMR90 cells were transfected with control or USP13 siRNA for 72 h, and then treated with TGF-β1 (10 ng/mL) for additional 6 h. Cell lysates were analyzed by immunoblotting with TXNIP and β-actin antibodies. (**D**) Intensities of TXNIP were quantified by ImageJ software and normalized to β-actin. *n* = 3. ** *p* < 0.01, compared to control. Shown are representative blots from three independent experiments.

**Figure 7.**
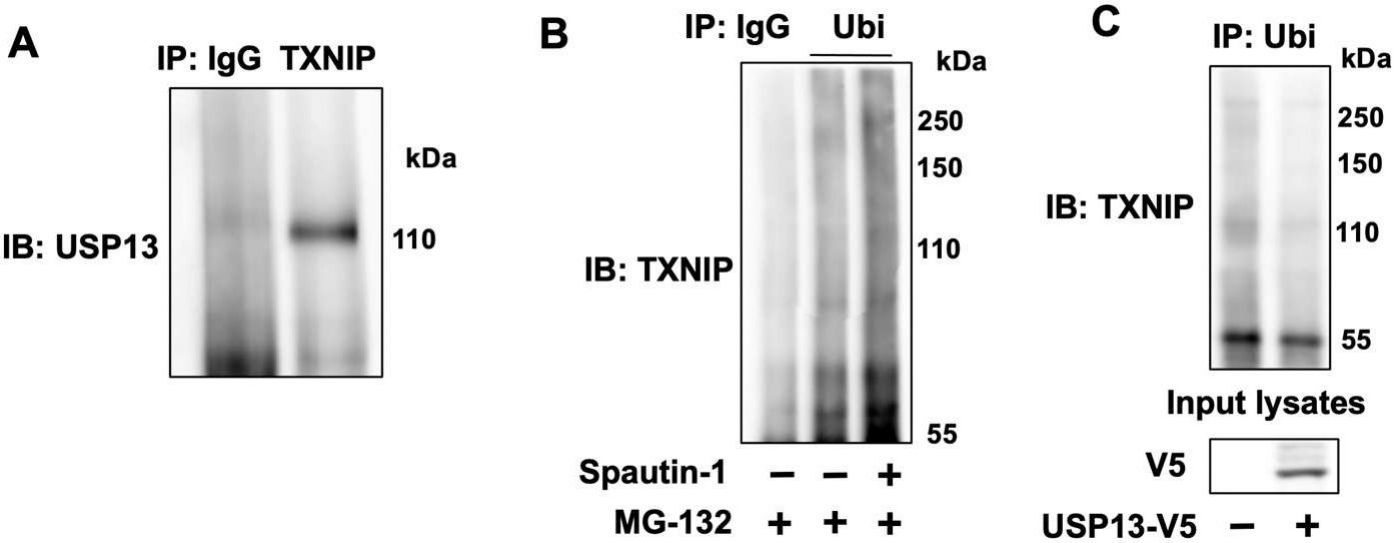
USP13 targets and deubiquitinates TXNIP. (**A**) IMR90 cell lysates were subjected to immunoprecipitation with rabbit IgG and TXNIP antibody, followed by immunoblotting analysis with USP13 antibody. (**B**) IMR90 cells were treated with MG-132 (20 μM, 1 h) prior to addition of spautin-1 (5 μM, 6 h). Denatured cell lysates were subjected to immunoprecipitation with IgG or Ubi antibody, followed by TXNIP immunoblotting. C. IMR90 cells were transfected with plasmids encoding USP13-V5 for 48 h, and then denatured cell lysates were subjected to immunoprecipitation with IgG or Ubi antibody, followed by TXNIP immunoblotting. Input cell lysates were analyzed by immunoblotting with V5 antibody.

While our previous study indicated that downregulation of USP13 reduced TXNIP levels [21], the role of USP13 in TGF-β1-induced TXNIPdegradation and its molecular mechanism have not been further revealed. The reduction of TXNIP after downregulation of USP13 was confirmed in HEK293 cells (Figure S3). Further, we found that overexpression of USP13 attenuated TGF-β1-induced TXNIP degradation (Figure 6A,B), while downregulation of USP13 promoted TXNIP degradation (Figure 6C,D), suggesting USP13 promotes TXNIP’s stabiliy. Given that S308A mutant preserved TXNIP stability when subjected to TGF-β1 treat,ent, we hypothesized that inhibiting USP13 may demonstrate less degradation than wild type when treated with a USP13 inhibitor. However, USP13 inhibitor spautin-1 reduced levels of both TXNIP wild type and S308A mutant (Figure S4), suggesting that USP13-regulated TXNIP stability is independent on TGF-β1 exposure. To investigate if USP13 stabilizes TXNIP by targeting it for deubiquitination, we performed immunoprecipitation. As shown in Figure 7A, USP13 was detected in the TXNIP antibody-precipitated complex, suggesting that USP13 is associated with TXNIP. Treatment with a TXNIP inhibitor, spautin-1, increased TXNIP polyubiquitination (Figure 7B), while overexpression of USP13 decreased TXNIP polyubiquitination (Figure 7C), suggesting USP13 targets and deubiquitinates TXNIP, leading to stabilization of TXNIP.

## 4. Discussion

Protein degradation plays a critical role in maintaining protein homeostasis under physiological conditions, while under pathological conditions, aberrant protein degradation may contribute to the initiation and development of diseases [22,23]. Deubiquitination reverses protein ubiquitination and stabilizes target proteins [24]. TXNIP exhibits various biological properties, including acting as a pro-oxidant and acitvating the NLRP3 inflammasome [11-13]. A recent study revealed that nuclear TXNIP regulates gene expression [12]. In the current study, we show that TXNIP displays an anti-fibrotic effect by attenuating TGF-β1-mediated signaling. TXNIP is degraded by the ubiquitin-proteasome system in the presence of TGF-β1. USP13 deubiquitinates and stabilizes TXNIP in lung fibroblasts. This is the first study to reveal that TXNIP stability is regulated by a deubiquitinase. The study may therefore provide a molecular basis to develop new small molecules to degrade TXNIP based on TXNIP/USP13 interaction.

Two independent studies demonstrated that TXNIP exhibits a pro-fibrotic property in renal fibrosis [25,26], but has been shown to exhibit an anti-fibrotic property in the liver [27]. TXNIP deficient mice display severe hypoglycemia and liver steatosis [28]. The role of TXNIP in pulmonary fibrosis has not been well studied. Han, et al. showed that TXNIP levels were elevated in a bleomycin-induced lung fibrosis model and TGF-β1-treated lung fibroblasts [29]. Our current results demonstrate the opposite phenomenon, indicating that TXNIP levels decreased in a bleomycin-induced lung fibrosis model and in response to TGF-β1. The conflicting results may have arisen due to differences in doses and sources of bleomycin used in the two studies. We feel confident in our conclusion as our results show that the effect of TGF-β1 on TXNIP degradation is consistent in various cell types including lung fibroblasts, epithelial cells, and HEK293 cells. Although it has been shown that knockdown of TXNIP attenuated TGF-β1-induced epithelial to mesenchymal transition (EMT) in kidney cells (HK-2 cells) [30], Masaki, S. et al. reported that downregulation of TXNIP exacerbated TGF-β1-induced epithelial to mesenchymal transition in A549 cells [31]. Our data also supports that TXNIP can inhibit TGF-β1 signaling in lung fibroblasts and epithelial cells. Taken together, the results indicate that the role of TXNIP in TGF-β1 signaling may differ between cell types.

Ubiquitination-mediated TXNIP degradation has been reported [15,16,20,32]. Phosphorylation is a modification known to trigger protein ubiquitination. Extracellular signal-regulated kinases (Erks) and AKT protein kinases phosphorylate TXNIP and promote its degradation [20,32]. In this study, we observed that ser 308 is a critical residue for TGF-β1-induced TXNIP degradation. Ser 308 phosphorylation under TGF-β1 exposure and identification of the kinase involved will be invesitgated in our future studies to further investigate the molecular mechanisms of TXNIP degradation. It is possible that ser 308 phosphorylation may increase TXNIP association with Itch E3 ligase, which has been shown to induce TXNIP degradation. In our previous study, we revealed a molecular mechanism by which phosphorylation modulates protein stability by determining target protein association with an E3 ligase or DUB. Phosphorylation increases Nedd4L E3 ligase association with lysophosphatidic acid receptor 1 (LPA1), and reduces deubiquitinase USP11 interaction with LPA1 [33]. In the current study, we show that USP13 targets and deubiquitinates TXNIP. In our future studies, we will investigate if ser 308 phosphorylation prevents USP13/TXNIP association. A recent study from Shi, et al. demonstrated that another DUB, USP5, stabilizes TXNIP in liver cells [34]. USP5 may therefore play a role in TGF-β1-induced TXNIP degredation as well.

Like other DUBs, USP13 regulates multiple proteins, such as Smad4 [35], Sigirr [36], MDM2 [37], STAT1 [38], and plays a role in a wide range of cellular functions. Here, we provide evidence showing that USP13 stabilizes TXNIP. Though TXNIP has an anti-TGF-β1 signaling effect in lung cells, USP13 has been shown to increase FN levels by stabilizing Smad4 [35], suggesting that the effect of USP13 on TGF-β1 signaling is complex. To avoid directly targeting USP13, it is important to develop a small molecule that may disrupt or promote USP13/TXNIP association, thereby promoting TXNIP degradation or stabilization. Thus, this study reveals a molecular basis for development of small molecules that can regulate TXNIP stability to treat diseases such as cancers and lung fibrosis.

## Supplementary Material

Supplementary Information
