# Supplementary material for: Molecular Regulation of Transforming Growth Factor-β1-induced Thioredoxin-interacting Protein Ubiquitination and Proteasomal Degradation in Lung Fibroblasts: Implication in Pulmonary Fibrosis
Source: J Respir Biol Transl Med. Author manuscript; Available in PMC 2024 Mar 25. (PMC10962057; doi:10.35534/jrbtm.2024.10002)
Supplement: Supplementary Information [file NIHMS1973559-supplement-Supplementary_Information.pdf]

Supplementary Information

Molecular Regulation of Transforming Growth Factor-β1-induced Thioredoxin-interacting Protein Ubiquitination and Proteasomal Degradation in Lung Fibroblasts: Implication in Pulmonary Fibrosis

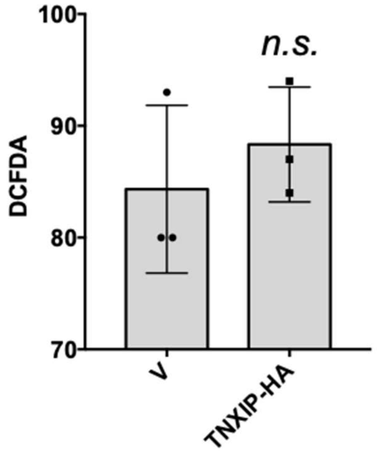

Figure S1. Overexpression of TXNIP has no changes in ROS production. IMR90 cells were transfected with plasmids encoding TXNIP-HA for 48 h. Cells were collected for ROS measurement with a fluorescent probe dichlorodihydrofluorescein diacetate (DCFDA). n = 3. n.s. compared to vehicle (V).

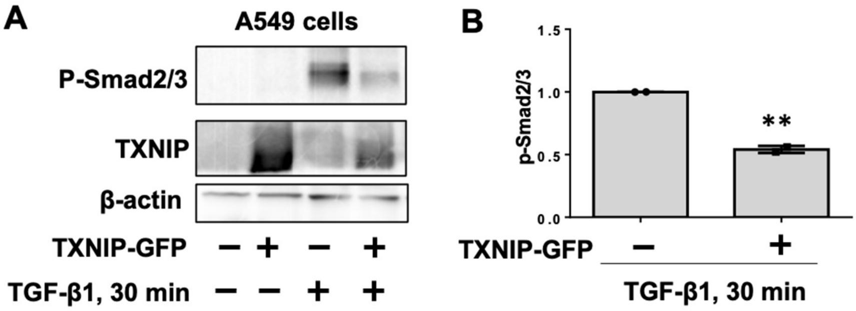

Figure S2. Overexpression of TXNIP attenuates TGF-β1-induced phosphorylation of Smad2/3 in A549 cells. **A.** A549 cells were transfected with plasmids encoding TXNIP-GFP for 48 h, then treated with TGF-β1 (10 ng/mL) for 30 min. Cell lysates were analyzed for immunoblotting with p-Smad2/3, TXNIP, and β-actin antibodies. B. Intensities of p-Smad2/3 were quantified with Image J software. n = 3. ** $p < 0.01$, compared to TGF-β1 alone. Shown were representative blots from three independent experiments.

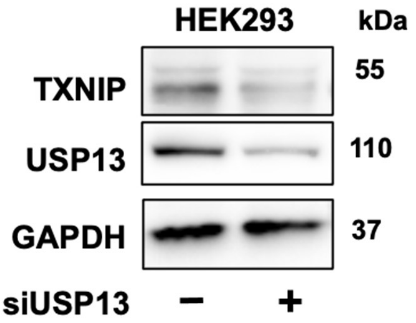

Figure S3. Downregulation of USP13 reduces TXNIP levels in HEK293 cells. HEK293 cells were transfected with control and USP13 siRNA for 72 h. Cell lysates were analyzed by immunoblotting with TXNIP, USP13, and GAPDH antibodies.

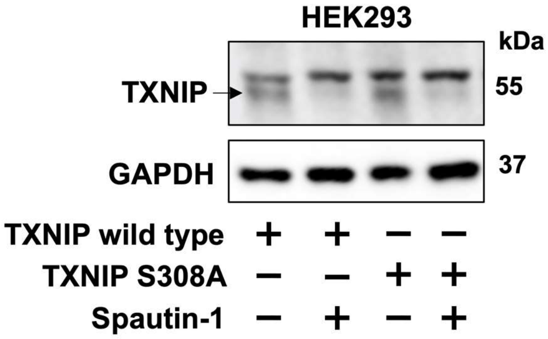

Figure S4. USP13 inhibitor spautin-1 reduces both levels of TXNIP wild type and S308A mutant. HEK293 cells were transfected with TXNIP-HA wild type or TXNIP S308A-HA mutant for 48 h, then cells were treated with spautin-1 (5 μM) for 6 h. Cell lysates were analyzed by immunoblotting with TXNIP and GAPDH antibodies. Shown were representative blots from four independent experiments.
